# Anti-Tumor Effects of Queen Bee Acid (10-Hydroxy-2-Decenoic Acid) Alone and in Combination with Cyclophosphamide and Its Cellular Mechanisms against Ehrlich Solid Tumor in Mice

**DOI:** 10.3390/molecules26227021

**Published:** 2021-11-20

**Authors:** Aishah E. Albalawi, Norah A. Althobaiti, Salma Saleh Alrdahe, Reem Hasaballah Alhasani, Fatima S. Alaryani, Mona Nasser BinMowyna

**Affiliations:** 1 Department of Biology, Faculty of Science, University of Tabuk, Tabuk 71491, Saudi Arabia; salrdahe@ut.edu.sa; 2 Department of Biology, College of Science and Humanities-Al Quwaiiyah, Shaqra University, Al Quwaiiyah 19257, Saudi Arabia; nalthobaiti@su.edu.sa; 3 Department of Biology, Faculty of Applied Science, Umm Al-Qura University, Makkah 21961, Saudi Arabia; rhhasani@uqu.edu.sa; 4 Department of Biology, Faculty of Sciences, University of Jeddah, Jeddah 21959, Saudi Arabia; fsalaryani@uj.edu.sa; 5 College of Applied Medical Sciences, Shaqra University, Shaqra 11961, Saudi Arabia; m.mwena@su.edu.sa

**Keywords:** cancer, treatment, royal jelly, natural products, breast cancer, in vivo

## Abstract

Queen bee acid or 10-hydroxy-2-decenoic acid (10-HDA) is one of the main and unique lipid components (fatty acids) in royal jelly. Previous studies have demonstrated that 10-HDA has various pharmacological and biological activities. The present study aims to evaluate the anti-tumor effects of 10-HDA alone and combined with cyclophosphamide (CP), as an alkylating agent which widely used for the treatment of neoplastic cancers, against the Ehrlich solid tumors (EST) in mice. Methods: A total of 72 female Swiss albino mice were divided into eight groups. EST mice were treated with 10-HDA (2.5 and 5 mg/kg) alone and combined with CP (25 mg/kg) orally once a day for 2 weeks. Tumor growth inhibition, body weight, the serum level of alpha-fetoprotein (AFP) and carcinoembryonic antigen tumor (CAE), liver and kidney enzymes, tumor lipid peroxidation (LPO) and nitric oxide (NO), antioxidant enzymes (e.g. glutathione reductase (GR), glutathione peroxidase (GPx), catalase enzyme (CAT)), tumor necrosis factor alpha level (TNF-α), and the apoptosis-regulatory genes expression were assessed in tested mice. Results: the findings exhibited that treatment of EST-suffering mice with 10-HDA at the doses of 2.5 and 5 mg/kg especially in combination with CP significantly (*p* < 0.001) decreased the tumor volume and inhibition rate, tumor markers (AFP and CEA), serum level of liver and kidney, LPO and NO, TNF-α level, as well as the expression level of Bcl-2 in comparison with the mice in the C2 group; while 10-HDA at the doses of 2.5 and 5 mg/kg especially in combination with CP significantly (*p* < 0.001) improved the level of antioxidant enzymes of GPx, CAT, and SOD and the expression level of caspase-3 and Bax genes. **Conclusions**: According to the results of the present investigations, 10-HDA at the doses of 2.5 and 5 mg/kg especially in combination with CP showed promising antitumor effects against EST in mice and can be recommended as a new or alternative anticancer agent against tumor; nevertheless, further investigations, particularly in clinical setting, are required to confirm these results.

## 1. Introduction

Cancer, with more than 30 different types, has been recently considered the principal cause of death around the world [1]; in 2018, it caused approximately 9.6 million deaths worldwide [2]. Cancer as a public health concern is described by unregulated multiplying of the body cells, due to loss of the cellular modulation and suppression of cell cycle development, subsequently provoking the formation of malignant tumors with the possibility of spreading and metastasizing to various organs [3,4].

Nowadays, a variety of strategies exist such as radiotherapy, surgery, and chemotherapy for management and treatment of cancers [5]. Considering chemotherapy, a number of synthetic drugs including antimetabolites drugs (e.g. methotrexate), passive compounds of DNA (e.g. doxorubicin and cisplatin), antitubulin drugs (e.g taxis), etc., are used for treating cancer [6,7]. However, these synthetic drugs are associated with some side effects and disadvantages such as gastrointestinal and kidney disorders, bone marrow suppression, fatigue, and also the resistance of cancer cells to common therapies [6,7]. Therefore, finding a new drug with high efficiency and low toxicity is one of the priorities of researchers in the field of cancer treatment.

Natural products have long been one of the most valuable sources of medicine due to their high availability, wide efficiency, and low toxicity [8]. Royal jelly (RJ) is a viscous substance secreted from the glands of mandibular and hypopharyngeal of *Apis mellifera* (worker bees). RJ, because of having various bioactive compounds such as lipids, protein, polyphenols, flavonoids, etc., has numerous pharmacological properties such as antioxidant, neurotrophic, anticancer, anti-inflammatory, antidiabetic, anti-lipidemic, antimicrobial, etc. [9,10]. The lipids make approximately 3 to 20% of the dry volume of RJ, whereas more than 90% of RJ lipids are identified the hydroxyl or dicarboxylic fatty acids [11]. (*E*)-10-hydroxy-2-decenoic acid (10-HDA) is one of the main and unique lipid components (fatty acids) in RJ [12,13]. Previous studies have demonstrated that 10-HDA have various pharmacological and biological activities such as anti-cancer, anti-inflammatory, immunomodulatory, and antimicrobial properties [14,15,16,17]. The present study aims to evaluate the anti-tumor effects of 10-HDA alone and combined with cyclophosphamide (CP), as an alkylating agent which widely used for the treatment of neoplastic cancers, against the Ehrlich solid tumors in mice.

## 2. Results and Discussion

Recently, studies have reported some limitations, disadvantages, and side effects of present drugs for treating cancer; therefore, finding a new drug with high efficiency and low toxicity is one of the priorities of researchers in the field of cancer treatment [5]. RJ as young worker bee’s secretion has potent nutritional and pharmacological properties due to possessing high amounts of proteins, lipids, carbohydrates, vitamins, etc. [11]. Chemical composition analysis of RJ displayed that free fatty acids, with 8–12 carbons (e.g., hydroxyl and dicarboxylic formulae) are the main lipids of dry RJ [11,12]. The present study aims to evaluate the anti-tumor effects of 10-HDA alone and combined with CP against the Ehrlich solid tumors in mice.

**Figure 1:**
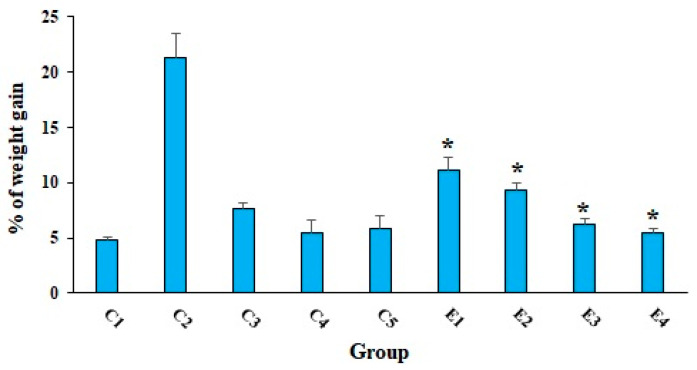
The mean of % of weight gains in EST-bearing mice treated with 10-HDA alone and in combination with cyclophosphamide (CP) when compared with non-EST and non-treated mice. Non-EST and non-treated mice (C1); EST mice receiving the normal saline (C2); EST mice treated with CP (50 mg/kg) intraperitoneally once a day for 3 days (C3); non-EST mice treated with 10-HDA 2.5 mg/kg orally once a day for 2 weeks (C4); non-EST mice treated with 10-HDA 5 mg/kg orally once a day for 2 weeks (C5); EST mice treated with 10-HDA 2.5 mg/kg orally once a day for 2 weeks (E1); EST mice treated with 10-HDA 5 mg/kg orally once a day for 2 weeks (E2); EST mice treated with 10-HDA 2.5 mg/kg + CP (25 mg/kg) once a day for 2 weeks (E3); EST mice treated with 10-HDA 5 mg/kg + CP (25 mg/kg) once a day for 2 weeks (E4). Data are expressed as the mean ± SD (n = 3). * *p* < 0.001 significant difference compared with C2 group.

As shown in Table 1, treatment of EST-suffering mice with 10-HDA especially in combined with CP significantly (*p* < 0.01) decreased the mean weight of tumors and the tumor inhibition rate in a dose-dependent response. The results exhibited that the tumor inhibition rates were 37.2, 57.7, 80.1, 89.7, and 59.3% after treatment of EST-suffering mice in the E1, E2, E3, E4, and C3 groups, respectively. Based on the obtained results, the combination of 10-HDA (2.5 and 5 mg/kg) and CP (50 mg/kg) exhibited significant antitumor effects (*p* < 0.05) on EST-suffering mice in comparison with CP alone. Considering the body weight evaluation, as displayed in Figure 1, EST-suffering mice treated with 10-HDA (2.5 and 5 mg/kg) especially in combined with CP (E3 and E4) exhibited a significant (*p* < 0.05) decrease in BW when compared with that of untreated EST mice in C2 control. 

Regarding the anticancer effects of 10-HDA in the studies conducted by Townsend et al., the results demonstrated that 10-HDA at the concentration of 1.5 mg/mL of cell suspension significantly inhibited the formation of tumor some cancer cells including mouse leukemia, 6CSHED lymphosarcoma, the TAS mammary carcinoma, and the Ehrlich carcinoma [18,19]. In addition, in the study conducted by Filipi et al. (2015), it has been proven that 10-HDA had a potent anti-proliferative effect at the concentration of 37.5 μmol/mL against human colorectal adenocarcinoma cells [20]. Recently, Lin et al. (2020) have revealed the promising in vitro anticancer effects of 10-HDA against human lung cancer cell lines of A549, NCI-H460, and NCI-H23with IC_50_ values of 22.68, 44.03, and 44.79 μM, respectively, whereas it had no significant cytotoxicity on IMR90 human normal lung fibroblasts [21].

It has been proven that one of the most important strategies for cancer treatment is inhibiting cancer cell migration; on the other hand, the TGF-β1 signaling pathway has been proven to modulate various physiologic processes, including proliferation, migration, and invasion of tumors [21]. Lin et al. (2020) have revealed that 10-HDA through downregulation of TGF-*β*1, SANI 1, GSK-3*β*, N-cadherin, and vimentin affects signal transduction pathways in A549 cells, so that the cells could not proliferate normally, limiting their range of activities, and thereby inhibiting the migration of lung cancer cells. They finally reported that 10-HDA displayed their anticancer effects through inducing ROS-mediated apoptosis in A549 human lung cancer cells by regulating the MAPK, STAT3, NF-κB, and TGF-β1 signaling pathways [21]. 

Studies have demonstrated the use of CP in therapy of solid tumors has been associated with some side effects such as suppression of bone marrow (leukopenia and thrombocytopenia), the impairment of organ functions, as well as the decline of the quality of life in patients [22]. Previous investigation showed that the use of antioxidant supplementation protects CP-induced toxicity; similarly, Khazaei et al (2019) have reported that RJ prevented the biochemical and histological damages induced by CP in male Wistar rats [23]. In addition, Fahmy et al. (2015) have revealed that combination of honey bee products (honey, royal jelly, and pollen grains) can be applied as a chemo-preventive agent for reducing the genotoxic side effects of the CP in Balb/c mice [24]. 

Considering the possible anticancer mechanisms of fatty acids, previous studies have demonstrated that these compounds displayed their anticancer role in the prevention and the development of cancers through some mechanisms such as including the unregulated cell signaling, suppression of cellular multiplying and differentiation, apoptosis induction, effect on cell membrane permeability, generation of reactive oxygen species (ROS), etc. [25]. Today, studies showed that adjustment of current anticancer drugs by combining these agents with some bioactive compounds can increase the tissue selectivity and may make chemotherapy considerably more effective with lower toxicity [26,27]. In this regard, a number of studies have reported the in vitro and in vivo promising effects of fatty acids which improved the efficacy of the antitumor drugs which enhance the tumor cell targeting selectivity and reduce the cytotoxic effects [25,28]. 

**Figure 2:**
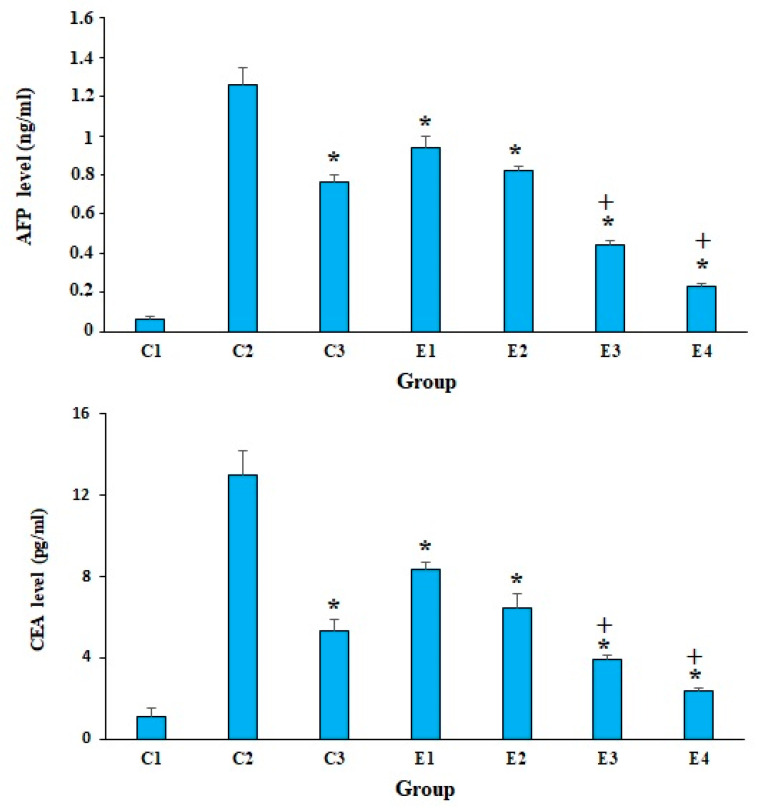
The serum level of alpha-fetoprotein (AFP) and carcinoembryonic antigen tumor (CEA) in EST-bearing mice treated with 10-HDA alone and in combination with cyclophosphamide (CP). Non-EST and non-treated mice (C1); EST mice receiving the normal saline (C2); EST mice treated with CP (50 mg/kg) intraperitoneally once a day for 3 days (C3); EST mice treated with 10-HDA 2.5 mg/kg orally once a day for 2 weeks (E1); EST mice treated with 10-HDA 5 mg/kg orally once a day for 2 weeks (E2); EST mice treated with 10-HDA 2.5 mg/kg + CP (25 mg/kg) once a day for 2 weeks (E3); EST mice treated with 10-HDA 5 mg/kg + CP (25 mg/kg) once a day for 2 weeks (E4). Data are expressed as the mean ± SD (*n* = 3). * *p* < 0.001 significant difference compared with C2 group; + *p* < 0.001 significant difference compared with C3 group.

According to studies, the elevated serum levels of some markers, such as AFP and CEA, indicate cancer-induced tissue damage [29]. The obtained results revealed that in the mice of control group of C2, the serum level of CEA and AFP was significantly elevated in comparison with the mice of C1 group. Nevertheless, the level of CEA and AFP was significantly (*p* < 0.001) decreased in the EST-suffering mice treated with 10-HDA at the doses of 2.5 and 5 mg/kg, especially in combined with CP, when compared with the mice of control groups (Figure 2). The obtained results also demonstrated that the combination of 10-HDA (2.5 and 5 mg/kg) and CP (25 mg/kg) exhibited significant reduction (*p* < 0.05) in the level of CEA and AFP of EST-suffering mice in comparison with CP alone.

**Figure 3:**
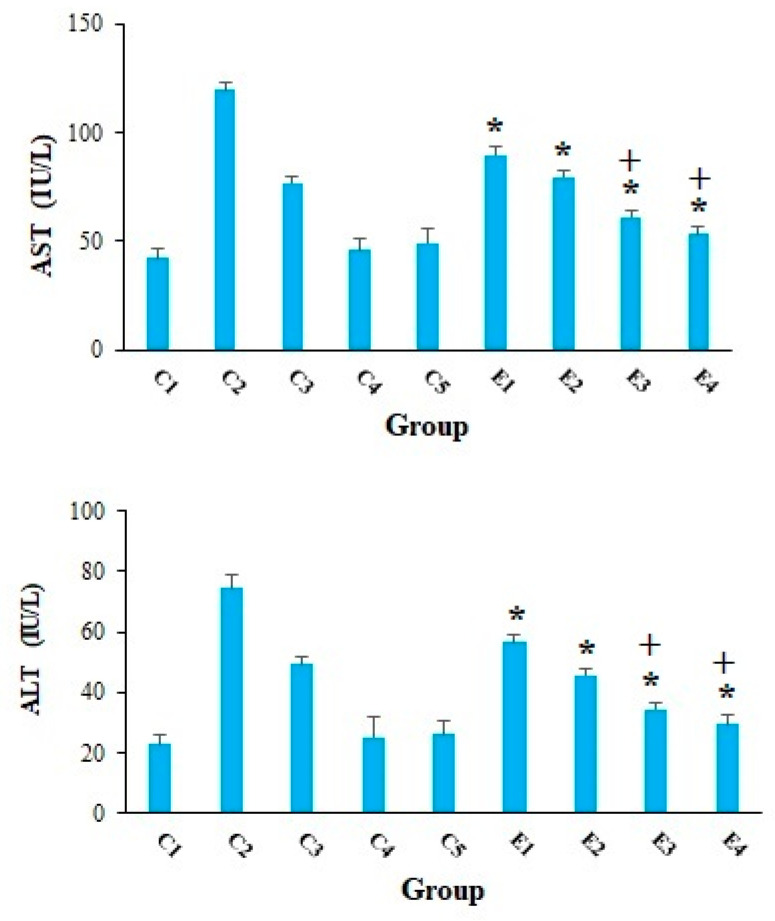
The serum level of alanine aminotransferase (ALT) and aspartate aminotransferase (AST) in EST-bearing mice treated with 10-HDA alone and in combination with cyclophosphamide (CP). Non-EST and non-treated mice (C1); EST mice receiving the normal saline (C2); EST mice treated with CP (50 mg/kg) intraperitoneally once a day for 3 days (C3); non-EST mice treated with 10-HDA 2.5 mg/kg orally once a day for 2 weeks (C4); non-EST mice treated with 10-HDA 5 mg/kg orally once a day for 2 weeks (C5); EST mice treated with 10-HDA 2.5 mg/kg orally once a day for 2 weeks (E1); EST mice treated with 10-HDA 5 mg/kg orally once a day for 2 weeks (E2); EST mice treated with 10-HDA 2.5 mg/kg + CP (25 mg/kg) once a day for 2 weeks (E3); EST mice treated with 10-HDA 5 mg/kg + CP (25 mg/kg) once a day for 2 weeks (E4). Data are expressed as the mean ± SD (*n* = 3). * *p* < 0.001 significant difference compared with C2 group; + *p* < 0.001 significant difference compared with C3 group.

**Figure 4:**
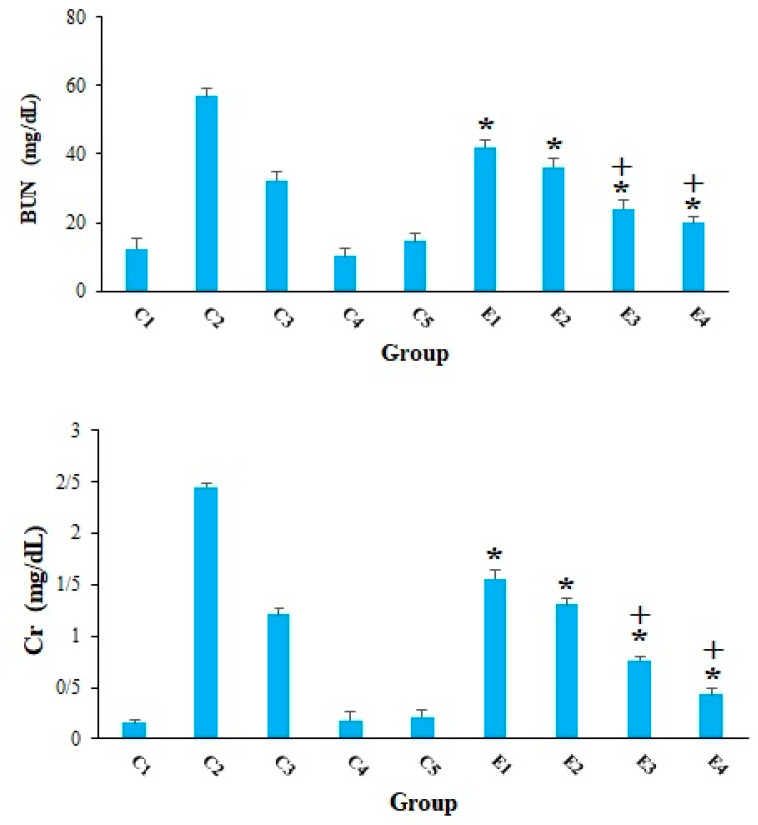
The serum level of blood urea nitrogen (BUN) and creatinine (Cr) in EST-suffering mice treated with 10-HDA alone and in combination with cyclophosphamide (CP). Non-EST and non-treated mice (C1); EST mice receiving the normal saline (C2); EST mice treated with CP (50 mg/kg) intraperitoneally once a day for 3 days (C3); non-EST mice treated with 10-HDA 2.5 mg/kg orally once a day for 2 weeks (C4); non-EST mice treated with 10-HDA 5 mg/kg orally once a day for 2 weeks (C5); EST mice treated with 10-HDA 2.5 mg/kg orally once a day for 2 weeks (E1); EST mice treated with 10-HDA 5 mg/kg orally once a day for 2 weeks (E2); EST mice treated with 10-HDA 2.5 mg/kg + CP (25 mg/kg) once a day for 2 weeks (E3); EST mice treated with 10-HDA 5 mg/kg + CP (25 mg/kg) once a day for 2 weeks (E4). Data are expressed as the mean ± SD (n = 3). * *p* < 0.001 significant difference compared with C2 group; + *p* < 0.001 significant difference compared with C3 group.

Cancer cells can have a detrimental effect on metabolism of liver and kidney cells, so that they enhance the serum levels of liver and kidney enzymes [30]. Our results, in agreement with other studies [31,32,33,34], revealed that the serum level of AST and ALT was remarkably elevated in the mice of C2 group, representing that EST triggered severe hepatocellular damage. However, in the EST-suffering mice treating with 10-HDA at the doses of 2.5 and 5 mg/kg especially in combined with CP, the level of ALT and AST was considerably (*p* < 0.001) declined compared with the mice of control groups (Figure 3). Figure 4 indicated that the serum level of BUN and Cr considerably raised in in the EST-suffering mice in control group of C2; conversely, treatment of the EST-suffering mice with 10-HDA at the doses of 2.5 and 5 mg/kg especially in combined with CP, the level of BUN and Cr was significantly (*p* < 0.001) declined compared with the mice of C2 group. Based on the obtained findings, the combination of 10-HDA (2.5 and 5 mg/kg) and CP (25 mg/kg) significantly (*p* < 0.05) reduced the serum level of liver and kidney enzymes in the EST-suffering mice, when compared with CP alone. Our results also demonstrated that treatment of healthy mice with 10-HDA at the doses of 2.5 and 5 mg/kg/day for two weeks did not show a significant change in serum level of liver and kidney enzymes in comparison with control group C1.

Considering the toxicity effects of 10-HDA, the results of the present investigation revealed that the oral administration of the 10-HAD at the doses of 2.5 and 5 mg/kg/day for 14 days has no significant toxicity effects on the serum level of liver and kidney enzymes as well as body weight in the healthy mice without EST. To the best of our knowledge, there are few documented studies on toxic effects of 10-HAD on animals. For instance, Weiser et al. (2017) have demonstrated that the administration of 10-HAD at the doses of 12–24 mg/kg/day in male Sprague-Dawley rats over more than 3 months increased body weight, and led to better maintenance of body weight with age. Furthermore, 10-HAD at the doses of 30–60 mg/kg/day in Balb/C mice for more than 3 months increased body weight, muscle mass, and adiposity in males, and increased bone density, but decreased adiposity, in females [35]. 

**Figure 5:**
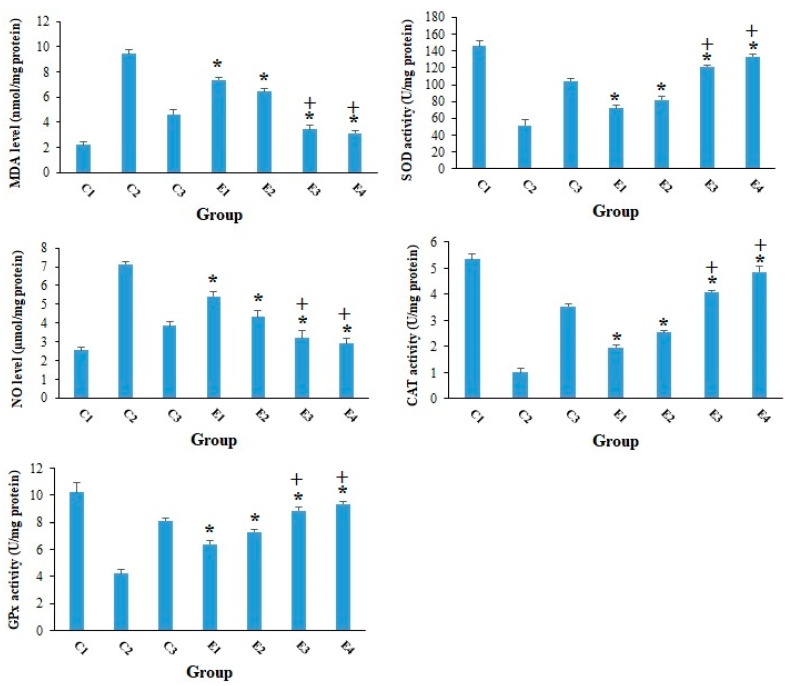
The tumor level of the malondialdehyde (MDA) and nitric oxide (NO) in EST-suffering mice treated with 10-HDA alone and in combination with cyclophosphamide (CP). Non-EST and non-treated mice (C1); EST mice receiving the normal saline (C2); EST mice treated with CP (50 mg/kg) intraperitoneally once a day for 3 days (C3); EST mice treated with 10-HDA 2.5 mg/kg orally once a day for 2 weeks (E1); EST mice treated with 10-HDA 5 mg/kg orally once a day for 2 weeks (E2); EST mice treated with 10-HDA 2.5 mg/kg + CP (25 mg/kg) once a day for 2 weeks (E3); EST mice treated with 10-HDA 5 mg/kg + CP (25 mg/kg) once a day for 2 weeks (E3). Data are expressed as the mean ± SD (*n* = 3). * *p* < 0.001 significant difference compared with C2 group; + *p* < 0.001 significant difference compared with C3 group.

According to previous studies, one of the most important factors in the development and progression of cancer is oxidative stress, which acts through enhancing mutations and damage in DNA, genome variation, and inhibition of cell multiplying, etc. [36]. On the other hand, reviews have revealed that antioxidant agents, particularly those originating from natural products, are potentially able to interfere with carcinogenesis and prevent human beings from compensations of oxidative stress [37]. As shown in Figure 5, the obtained findings showed that although the tumor level of MDA and NO was significantly raised in the mice of C2 group, the level of GPx, CAT, and SOD was considerably reduced. In opposition, 10-HDA at the doses of 2.5 and 5 mg/kg especially in combined with CP considerably (*p* < 0.01) declined the expansion in the LPO and NO as well as raised (*p* < 0.05) the level of GPx, CAT, and SOD. Consistent with our results, Sugiyama et al. (2013) demonstrated that 10-HDA suppressed the LPS-induced NO production through inhibiting NF-κB activation [38]. 

**Figure 6:**
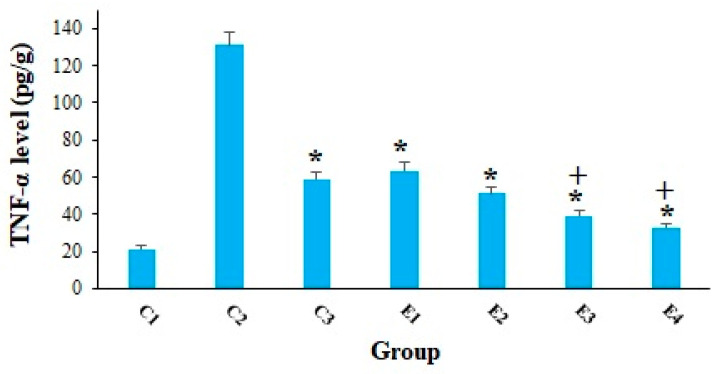
The tumor level of the TNF-αin EST-bearing mice treated with 10-HDA alone and in combination with cyclophosphamide (CP). Non-EST and non-treated mice (C1); EST mice receiving the normal saline (C2); EST mice treated with CP (50 mg/kg) intraperitoneally once a day for 3 days (C3); EST mice treated with 10-HDA 2.5 mg/kg orally once a day for 2 weeks (E1); EST mice treated with 10-HDA 5 mg/kg orally once a day for 2 weeks (E2); EST mice treated with 10-HDA 2.5 mg/kg + CP (25 mg/kg) once a day for 2 weeks (E3); EST mice treated with 10-HDA 5 mg/kg + CP (25 mg/kg) once a day for 2 weeks (E3). Data are expressed as the mean ± SD (*n* = 3). * *p* < 0.001 significant difference compared with C2 group; + *p* < 0.001 significant difference compared with C3 group.

Cancer cells have the ability to cause tissue damage and hypoxia in various organs in a variety of ways such as (i) mechanical harm of the tumor and (ii) provoking the secretion of some pro-inflammatory cytokines [39]. As shown in Figure 6, the obtained results confirmed that the level of TNF-α in the EST-suffering mice in C2 group was considerably (*p* < 0.001) raised; nevertheless, treatment of the EST-suffering mice with 10-HDA at the doses of 2.5 and 5 mg/kg especially in combination with CP, significantly (*p* < 0.05) declined the level of TNF-α in mice. Similarly, Yang et al (2018) have reported that the production of TNF-α was significantly decreased in human colon cancer cells (WiDr cells) after in vitro treatment by 10-HDA at the concentration of 3 mM, which indicated the promising anti-inflammatory activity in WiDr cells [40]. 

Programmed cell death, also called apoptosis, is considered as one of the important processes involved in living organisms’ biological development, and in the situation of irregular and abnormal activity, it can result in some diseases [41]. It has long been proven that the inhibition of apoptosis is crucial in tumorigenesis and cancers which is important for cancer cells to remain their uncontrollable multiplying. Therefore, induction and promotion of apoptosis is a standard goal to find new anti-cancer agents [42].

**Figure 7:**
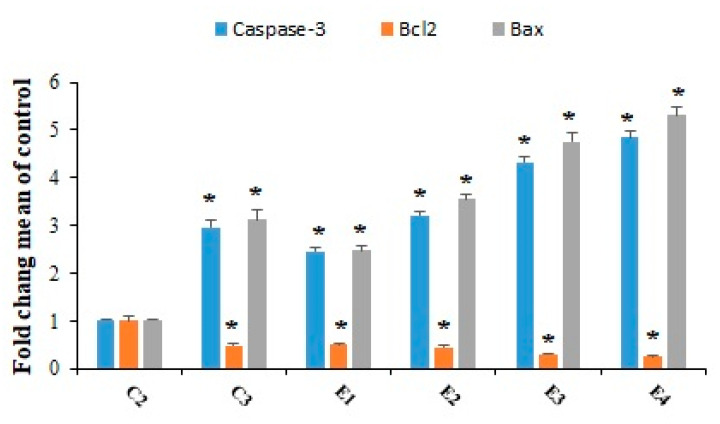
The expression level of the caspase-3, Bcl-2, and Bax genes in EST-bearing mice treated 10-HDA alone and in combination with cyclophosphamide (CP). EST mice receiving the normal saline (C2); EST mice treated with CP (50 mg/kg) intraperitoneally once a day for 3 days (C3); EST mice treated with 10-HDA 2.5 mg/kg orally once a day for 2 weeks (E1); EST mice treated with 10-HDA 5 mg/kg orally once a day for 2 weeks (E2); EST mice treated with 10-HDA 2.5 mg/kg + CP (25 mg/kg) once a day for 2 weeks (E3); EST mice treated with 10-HDA 5 mg/kg + CP (25 mg/kg) once a day for 2 weeks (E3). Data are expressed as the mean ± SD (*n* = 3). * *p* < 0.001 significant difference compared with C2 group.

Figure 7 exhibited that the expression level of caspase-3 and Bax genes was significantly (*p* < 0.001) up-regulated in tumor tissues, after treatment of the EST-suffering mice with 10-HDA at the doses of 2.5 and 5 mg/kg especially in combination with CP. The findings of quantitative real-time PCR also displayed that the expression level of Bcl2 was considerably (*p* < 0.05) downregulated in the tumor after treatment of the EST-suffering mice with 10-HDA at the doses of 2.5 and 5 mg/kg especially in combined with CP. Considering the effects of 1-HDA on apoptosis induction, in the study conducted by Lin et al. [24], the results showed that 10-HDA induced the apoptosis in human lung cancer cell lines (A549 cells) through the modulating mitochondrial-associated apoptosis, and caused cell cycle arrest at the G0/G1 phase in a time-dependent manner.

## 3. Materials and Methods

### 3.1. 10-HAD

**Figure 8:**
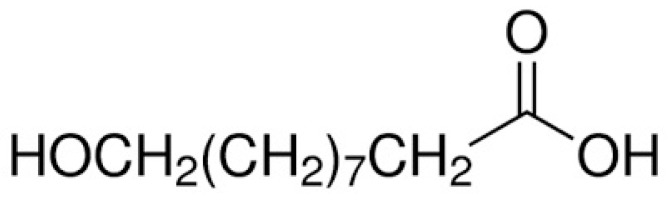
Chemical formula of queen bee acid (10-hydroxy-2-decenoic acid, C_10_H_18_O_3_).

Here, we prepared the 10-HAD with chemical formula of C_10_H_18_O_3_ (Figure 8) (purity > 99%) from Sigma Chemical (St. Louis, MO, USA).

### 3.2. Animals

To determine the animal model of EST, 72 female Swiss albino mice with an average weight of 25–20 g and 42–56 days old were used. All stages of this study were done in accordance with the recommendations of the Guide for Care and Use of Laboratory Animals of the National Institutes of Health. In addition, the study was accepted by the ethical committee of Shaqra University, Saudi Arabia (No. 20201123).

### 3.3. The Ehrlich Ascites Tumor (EAT) Cell Line and Induction of EST in Mice

We provided the Ehrlich ascites tumor (EAT) cell line from the American Type Tissue Culture Collection (Manassas, ATCC, USA); then we adjusted the cells into 2.5 × 10^6^ cells /mL in sterile saline solution using a Neubauer hemocytometer. Finally, in order to induce EAT in mice, we injected 200 µL of cells (2.5 × 10^6^ cells/mL) intramuscularly into the right thigh of mice.

### 3.4. Study Design 

The treatment of mice in the experimental group was started one week after of EST inoculation. Mice were divided into two main groups, including control group (C) with 40 mice in five sub-groups (C1, C2, C3, C4, C5) and experimental group with 32 mice in three sub-groups (E1, E2, E3, E4) including:

Non-EST and non-treated mice (C1) 

EST mice receiving the normal saline (C2)

EST mice treated with CP (50 mg/kg) itraperitoneally once a day for 3 days (C3)

Non-EST mice treated with 10-HDA 2.5 mg/kg orally once a day for 2 weeks (C4)

Non-EST mice treated with 10-HDA 5 mg/kg orally once a day for 2 weeks (C5) 

EST mice treated with 10-HDA 2.5 mg/kg orally once a day for 2 weeks (E1)

EST mice treated with 10-HDA 5 mg/kg orally once a day for 2 weeks (E2) 

EST mice treated with 10-HDA 2.5 mg/kg/day + CP (25 mg/kg/day) for 2 weeks (E3)

EST mice treated with 10-HDA 5 mg/kg/day+ CP (25 mg/kg/day) for 2 weeks (E4)

### 3.5. Blood and Tumor Sampling 

At the end of the experiment (day 21), mice were euthanized (by decapitation under intraperitoneal injection with sodium pentobarbital), their abdomen cavities opened, and blood samples were poised from the animal’s heart. Then the samples were centrifuged at 6000 rpm for 10 min and the collected sera were detached and kept at −80 °C until testing. After the tumors were removed aseptically, they were weighed and their dimensions recorded. In the last step, tumors were similarly divided into two parts: the first part was kept in −80 °C for molecular tests, and the second part was kept at −20 °C for other trials.

### 3.6. Tumor Growth Inhibition

By calculating the tumor volume (TV) and tumor growth inhibition rate (TGIR), the antitumor activity of 10-HDA alone and in combination with the CP was calculated. TV by the Vernier caliper after the 21st day of the treatment by the equation TV (mm^3^) = $4\pi {\left(\frac{A}{2}\right)}^{2}\times \left(\frac{B}{2}\right)$ was deliberated, where A and B are the minor and major tumor axes. The method of calculating TGIR is according to the following formula [43]:

(1) $$\mathrm{TGIR}\;(\%)=\frac{\left(The\;mean\;tumor\;weight\;of\;control\;group\;-\;the\;mean\;tumor\;weight\;of\;treated\;group\right)}{\left(The\;mean\;tumor\;weight\;of\;control\;group\right)}\times 100$$

### 3.7. Body Weight (BW) Changes 

All groups of animals were weighed on day 7 and day 21. The percentage weight gain was determined using the following equation [43]:

(2) $$\%\mathrm{weight}\;\mathrm{gain}=\left[\frac{\mathrm{Mice}\;\mathrm{weight}\;\mathrm{on}\;19\mathrm{th}\;\mathrm{day}}{\mathrm{Mice}\;\mathrm{weight}\;\mathrm{on}\;\mathrm{day}\;0}\right]-1\times 100\;$$

### 3.8. Evaluating the Tumor Markers

We determined the serum level of alpha-fetoprotein (AFP), according to the manufacturer’s instructions using an automated quantitative enzyme-linked fluorescent assay (ELFA) using mini-VIDAS® AFP (bioMérieux, Marcyl’Etoile, France). Additionally, the serum level of carcinoembryonic antigen tumor (CAE) was assessed by the quantitative sandwich immunoassay, MyBio-Source Mouse Carcinoembryonic Antigen Elisa Kit (MyBio-Source, San Diego, CA, USA).

### 3.9. Evaluation of Serum Levels of Liver Enzymes

The serum levels of some liver enzymes such as alanine aminotransferase (ALT) and aspartate aminotransferase (AST) were concluded using the commercial diagnostic kits (Roche, Germany). This was done to evaluate liver function after TBME treatment [44]. 

### 3.10. Evaluation of Serum Levels of Kidney Enzymes

After treatment mice in the tested groups, for evaluation of the function of kidney, the serum levels of creatinine (Cr) and blood Urea Nitrogen (BUN) were studied using the commercial diagnostic kits (Roche, Germany) [44].

### 3.11. Evaluation of the Oxidative Stress Markers 

In order to examine the oxidative stress (lipid peroxidation (LPO) and nitric oxide (NO) level, tumor homogenates were deliberated by biodiagnostic analysis kits along with the malondialdehyde (MDA) production through the thiobarbituric acid (TBA) technique [45]; furthermore, according to method described by Green et al. NO production was determined in the tumor suspension [46].

### 3.12. Evaluation of the Antioxidant Enzymes

To determine the levels of some effective enzymes in antioxidant mechanisms such as glutathione reductase (GR), glutathione peroxidase (GPx), catalase enzyme (CAT), and superoxide dismutase enzyme activity (SOD), we used the commercial kits based on the method defined by Ellman [47], Luck [48], and Sun et al. [49], respectively.

### 3.13. Measuring the Tumor Necrosis Factor Alpha Level (TNF-α)

The level of TNF-α in tumor homogenates were evaluated by the mice TNF-α ELISA kit (ab100747; Abcam, Cambridge, UK) according to the manufacturer’s procedure.

### 3.14. Evaluating the Apoptosis-Regulatory Genes Expression

In this study, the apoptosis-regulatory gene expressions on some apoptosis involving genes such as caspase-3, Bcl2, and Bax were evaluated by quantitative real-time PCR. Firstly, total RNA of tumor tissue was extracted using a RNeasy tissue kit (Qiagen, Germany) in accordance with the company orders. Then, cDNA synthesis was produced using random primers which were used for the complementary DNA (cDNA) synthesis based on the manufacturer’s recommendations. After completing this step, cDNA was applied for conventional PCR reaction analysis or real-time PCR through SYBR green. The thermal profile of response was 95 °C for 8 min, 40 cycles of 95 °C for 10 s, and 56 °C for 30 s, respectively. Lastly, the ΔCt was deliberated by means of the iQTM5 optical system software (Bio-Rad, Hercules, CA, USA). β-actin was applied as a housekeeping gene and normalization control. The oligonucleotide primers which were used for real-time PCR are shown in Table 2 [50].

### 3.15. Statistical Analysis

All data were showed as the means ± standard deviation. SPSS statistical software version, 22.0 (SPSS Inc., Chicago, IL, USA) were applied for data analysis. One-way ANOVA with Tukey’s *post-hoc* test was used to assess differences between experimental groups.

## 4. Conclusions

According to the results of the present investigations, 10-HDA at the doses of 2.5 and 5 mg/kg especially in combination with CP showed promising antitumor effects against EST in mice and can be recommended as a new or alternative anticancer agent; nevertheless, further investigations, particularly in a clinical setting, are required to confirm these results.

## Figures and Tables

**Table 1:** Effect of various doses of 10-HDA alone and in combination with cyclophosphamide (CP) on tumor volume and tumor inhibition rate in EST mice. (C2) EST mice receiving the normal saline; (C3) EST mice treated with CP (50 mg/kg) intraperitoneally once a day for 3 days (C3). Data are expressed as the mean ± SD (*n* = 3).

Group	Tumor Volume (g)	% of Inhibition
10-HDA 2.5 mg/kg	1.96 ± 0.083	37.2
10-HAD 5 mg/kg	1.32 ± 0.13	57.7
10-HAD 2.5 mg/kg + CP (25 mg/kg)	0.62 ± 0.031	80.1
10-HAD 5 mg/kg + CP (25 mg/kg)	0.32 ± 0.024	89.7
Control C2	3.12 ± 0.12	-
Control C3	1.27 ± 0.03	59.3

**Table 2:** Sequence of primers of used for real-time PCR.

Amplicon	Primers	Sequence (5′–3′)
Bax	F R	GGCTGGACACTGGACTTCCT GGTGAGGACTCCAGCCACAA
Bcl-2	F R	CATGCCAAGAGGGAAACACCAGAA GTGCTTTGCATTCTTGGA TGAGGG
Caspase-3	F R	TTCATTATTCAGGCCTGCCGAGG TTCTGACAGGCCATGTCATCCTCA
β-actin	F R	GTGACGTTGACATCCGTAAAGA GCCGGACTCATCGTACTCC

## Data Availability

All data generated or analyzed during this study are included in this published article.

## Sample Availability

Samples of the compounds of royal jelly are available from the authors.
